# etymologia: *Kaposi* [kah′po-she, kap′o-sē] *sarcoma*

**DOI:** 10.3201/eid1504.999999

**Published:** 2009-04

First described by dermatologist Moritz Kaposi (1837–1902) at the University of Vienna in 1872. Dr Kaposi’s last name was originally Kohn, but to distinguish himself from other physicians of the same name, he chose a new name in honor of the Kapos River, near his birthplace, Kaposvár, Hungary. The condition he described, Kaposi sarcoma, is a malignant tumor of the lymphatic endothelium, characterized by bluish-red cutaneous nodules. Human herpesvirus 8 has been implicated in its etiology.

